# Oncogenic Effect of the Novel Fusion Gene *VAPA-Rab31* in Lung Adenocarcinoma

**DOI:** 10.3390/ijms20092309

**Published:** 2019-05-10

**Authors:** Daseul Yoon, Kieun Bae, Jin-Hee Kim, Yang-Kyu Choi, Kyong-Ah Yoon

**Affiliations:** 1 College of Veterinary Medicine, Konkuk University, Seoul 05029, Korea; yds3123@naver.com (D.Y.); bkieun@naver.com (K.B.); yangkyuc@konkuk.ac.kr (Y.-K.C.); 2 College of Health Science, Cheongju University, Cheongju 28503, Korea; jinheekim@cju.ac.kr

**Keywords:** VAPA-Rab31, gene fusion, tumorigenicity, lung adenocarcinoma

## Abstract

Fusion genes have been identified as oncogenes in several solid tumors including lung, colorectal, and stomach cancers. Here, we characterized the fusion gene, *VAPA-Rab31*, discovered from RNA-sequencing data of a patient with lung adenocarcinoma who did not harbor activating mutations in *EGFR*, *KRAS* and *ALK*. This fusion gene encodes a protein comprising the N-terminal region of vesicle-associated membrane protein (VAMP)-associated protein A (VAPA) fused to the C-terminal region of Ras-related protein 31 (Rab31). Exogenous expression of *VAPA-Rab31* in immortalized normal bronchial epithelial cells demonstrated the potential transforming effects of this fusion gene, including increased colony formation and cell proliferation in vitro. Also, enhanced tumorigenicity upon VAPA-Rab31 was confirmed in vivo using a mouse xenograft model. Metastatic tumors were also detected in the liver and lungs of xenografted mice. Overexpression of *VAPA-Rab31* upregulated anti-apoptotic protein Bcl-2 and phosphorylated CREB both in cells and xenograft tumors. Reduced apoptosis and increased phosphorylation of CREB and Erk were observed in *VAPA-Rab31*-overexpressing cells after bortezomib treatment. Elevated Bcl-2 level via activated CREB contributed to the resistance to the bortezomib-induced apoptosis. Our data suggest the oncogenic function of the novel fusion gene *VAPA-Rab31* via upregulated Bcl-2 and activated CREB in lung cancer.

## 1. Introduction

Lung cancer is a common cancer and the leading cause of cancer death worldwide [1,2]. Lung adenocarcinoma is the predominant histologic type in non-small cell lung cancer, the majority of diagnosed lung cancers [3]. Lung adenocarcinoma can be classified according to the presence of major driver mutations in *EGFR*, *KRAS*, *ALK*, and *BRAF*, which confer clinical benefits to the subset of patients harboring them [4,5,6]. The fusion gene of *EML4-ALK* was discovered in lung adenocarcinoma in 2007 [7]; since then, fusion alterations of *ALK*, *RET*, and *ROS1* genes have been found in about 4–7% of patients with lung adenocarcinoma [8,9,10]. The fusions of these genes have been identified as novel drivers demonstrating oncogenic features. Fusion genes encoding tyrosine kinase provided enhanced sensitivity to selective inhibitors. For example, crizotinib has been demonstrated to be an effective targeted therapy in patients with lung adenocarcinoma harboring *ALK* or *ROS1* fusions [11,12]. Genetic rearrangements of *RET* or fibroblast growth factor receptors (*FGFR*) are rare, but can provide clinical benefits for the patients with these mutations. Therefore, the discovery of novel gene fusions and identification of their oncogenic features are important to elucidate novel targets. Here, we describe the identification of a fusion gene comprising part of the coding regions of *Rab31* and *VAMP-associated protein A* (*VAPA*) through RNA-sequencing in patients with lung adenocarcinoma.

*Rab31* (NM_006868) gene is a member of the RAS oncogene family that is located on chromosome 18 (18p11.22). Rab31 protein localizes in the cytosol, Golgi apparatus and endosomes, where it functions, through its GTP-binding activity, as one of the key regulators of intracellular membrane trafficking, from the formation of vesicles to their fusion with membranes [13,14]. The promotive effect of Rab31 on tumor progression has been reported in several kinds of cancers [15]. In glioblastoma, Rab31 induced cell proliferation by activating G1/S checkpoint transition and the PI3K/Akt pathway [16]. Rab31 was found to be overexpressed in estrogen receptor-positive breast cancer and enhanced proliferation of breast cancer cells [17]. Additionally, elevated *Rab31* expression was associated with poor prognosis in hepatocellular carcinoma through inhibition of apoptosis via the Bcl-2/Bax pathway [18]. *VAPA*, in turn, has been known to be involved in vesicle trafficking, membrane fusion, and cell motility [19].

In this study, we identified the fusion gene *VAPA-Rab31* in a patient with lung adenocarcinoma who did not harbor any known driver mutations in *EGFR*, *KRAS*, and *ALK*. Bronchial epithelial cells that overexpressed the fusion protein demonstrated enhanced proliferation and tumorigenicity. Our data suggest that the fusion gene *VAPA-Rab31* is a potential oncogene of lung cancer.

## 2. Results

### 2.1. Identification of the VAPA-Rab31 Fusion Gene in a Patient with Lung Adenocarcinoma

**Figure 1:**
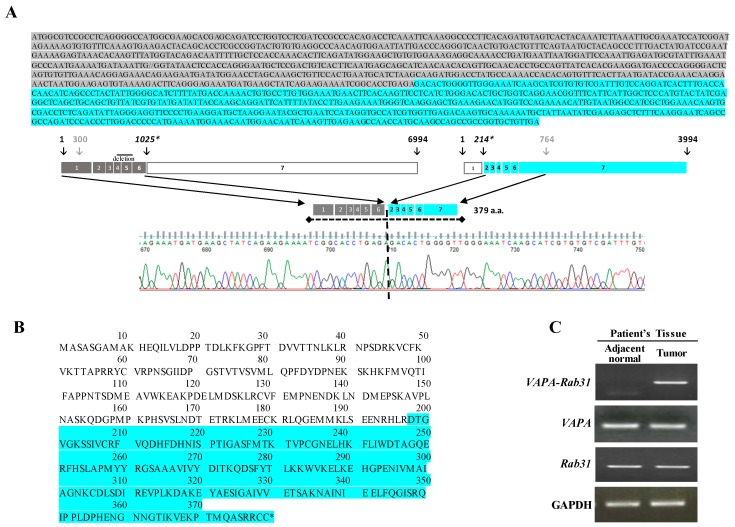
The *VAPA-Rab31* fusion gene is highly expressed in tumor tissue of a patient with lung adenocarcinoma. (**A**) The *VAPA* and *Rab31* exon structures are indicated. The schematic representation of the *VAPA-Rab31* fusion gene, with the exons from each gene, is also shown. The breakpoint of the fusion gene was confirmed by Sanger sequencing chromatogram. (**B**) Amino acid sequences of fusion gene: different colors are used for the two gene product (grey: VAPA; blue: Rab31). (**C**) The overexpression of *VAPA-Rab31* was confirmed by RT-PCR in the patient’s tumor tissue.

A fusion transcript of *Rab31* and *VAPA* was detected from RNA-sequencing data of a Korean patient with lung adenocarcinoma. The patient did not harbor any activating mutation of *EGFR*, *KRAS*, or *ALK* genes; he was diagnosed as poorly differentiated lung adenocarcinoma stage 2B at the age of 51 and was a light smoker that used one pack per year. The chimeric *VAPA-RAB31* mRNA encoded a fusion protein of 379 amino acids including exon 1 to 6 of *VAPA* and exon 2 to 7 of *Rab31*, in frame; the junction between the two genes was confirmed by Sanger sequencing analysis (Figure 1A,B). *VAPA-Rab31* fusion was highly expressed only in the tumor of the patient, and not in the matched normal tissue as confirmed by RT-PCR (Figure 1C). An intrachromosomal fusion of *VAPA-Rab31* was also detected in a case of hepatocellular carcinoma from The Cancer Genome Atlas (TCGA) database (www.tumorfusions.org). Another fusion transcript of *Rab31* was detected in a patient with invasive breast carcinoma and was originated by the fusion form of *DLGAP1-Rab31* (Table S1). The tumorigenic effect of VAPA-Rab31 has not been evaluated before; therefore, we decided to further analyze it.

### 2.2. Overexpression of VAPA-Rab31 Increases Colony Formation and Upregulates Bcl-2 Expression

**Figure 2:**
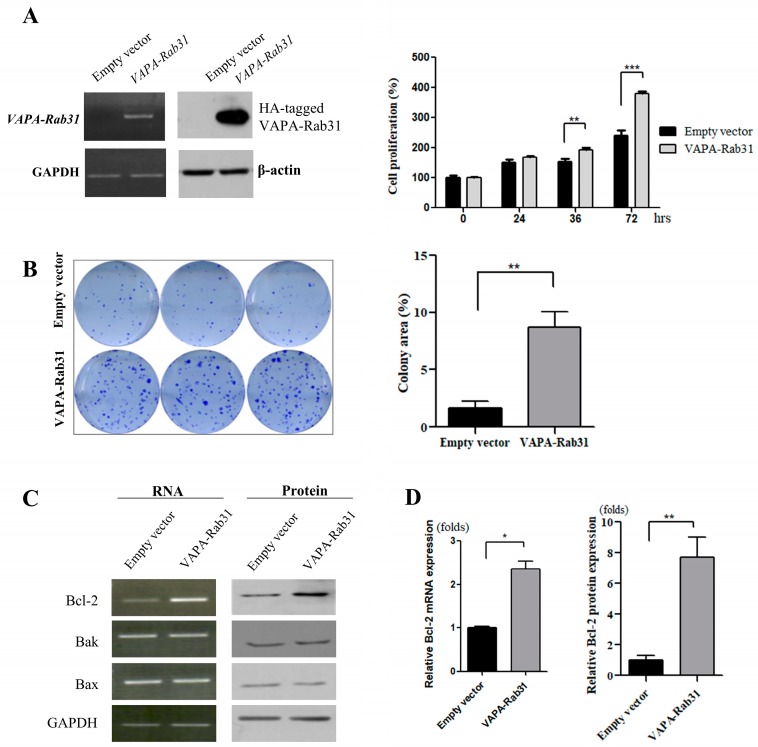
Overexpression of *VAPA-Rab31* fusion increases proliferation, colony forming activity, and Bcl-2 expression. (**A**) Lentivirus-mediated overexpression of *VAPA-RAb31* in Beas-2B cells was confirmed by RT-PCR and immunoblot assay detecting HA tag. Glyceraldehyde-3-phosphate dehydrogenase (GAPDH) and β-actin was used as control. Bar graphs indicated the relative cell numbers as the mean percentage (± standard error, SE) at the indicated time; the experiments were performed in triplicates. (** *p* < 0.01, *** *p* < 0.005) (**B**) *VAPA-Rab31*-overexpressing cells and control cells were cultured to compare colony forming activity as demonstrated as representative data. Bar graphs compare the area of colonies that measured using ImageJ software. Data are expressed as means ± SE from three independent experiments and a significant result is marked with ** (*p* value < 0.01). (**C**) Expression of Bcl-2, Bak and Bax were examined by RT-PCR and Western blotting. (**D**) The graph indicates the fold change of Bcl-2 expression relative to the expression in control cells. Statistical differences are marked with * (*p* < 0.05), and ** (*p* < 0.01), respectively.

To evaluate the effect of the fusion gene *VAPA-Rab31*, we stably overexpressed it in a human normal bronchial epithelial cell line (Beas-2B) using lentiviral vectors. As shown by the cell growth rate, the overexpression of *VAPA-Rab31* promoted the growth of Beas-2B cells (Figure 2A). Colony formation was also enhanced in *VAPA-Rab31*-overexpressing cells compared with control cells, overexpressing the empty vector (*p* < 0.01) (Figure 2B). Additionally, RT-PCR and immunoblot analyses showed that *VAPA-Rab31* overexpressing cells had higher levels of Bcl-2 than the control cells (Figure 2C,D).

### 2.3. VAPA-Rab31 Fusion Induces Resistance to Apoptosis

**Figure 3:**
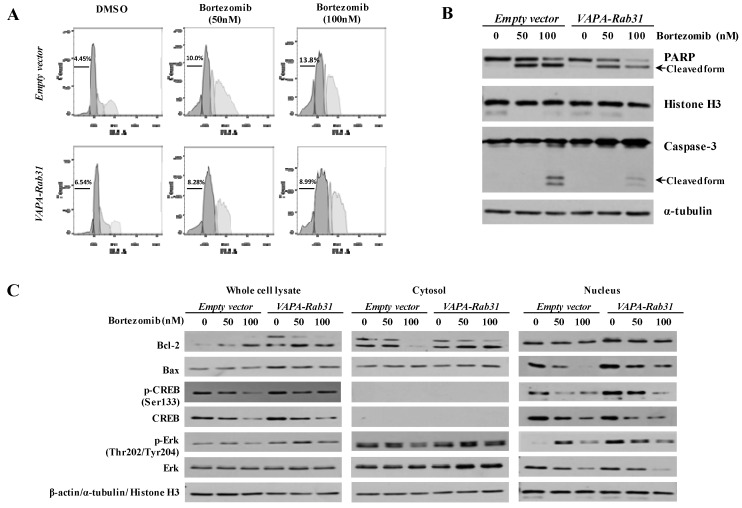
The *VAPA-Rab31* fusion gene induced the resistance to apoptosis. (**A**) Cell cycle was analyzed in 48 h after bortezomib treatment at the indicated concentration. The percentage of cells in sub-G1 phase (%) was indicated. (**B**) Cleavage of PARP and Caspase-3 were examined as the apoptotic markers by western blotting at 48 h after bortezomib treatment. (**C**) Protein expression was examined in whole cell lysates, cytosolic fractions, and nuclear fractions. Phosphorylated CREB and CREB were highly expressed in the nuclear fractions and did not detected in the cytosolic fractions. β-actin, α-tubulin, and histone H3 were used as loading control for whole cell lysates, cytosolic fraction, and nuclear fraction, respectively.

To examine the effect of VAPA-Rab31 on apoptosis, the apoptotic markers were compared after induction of apoptosis through bortezomib treatment. Cell cycle analysis revealed that the proportion of sub-G1 phase cells was smaller in the *VAPA-Rab31*-overexpressing cells than in the control cells (Figure 3A). The cleaved PARP and active Caspase-3 were strongly induced in the control cells after bortezomib treatment. However, the cleavage of PARP and the active form of Caspase-3 in VAPA-Rab31-overexpressing cells was not induced as much as the control cells after bortezomib treatment indicating reduced apoptosis (Figure 3B). The levels of relevant proteins and their phosphorylation were examined in cytosol, nucleus and whole cells. In *VAPA-Rab3*-expressing cells, Bcl-2 protein and phosphorylated CREB at serine 133 (p-CREB) were retained upregulation even after bortezomib treatment. Phosphorylated Erk (Thr202/Tyr204) was also increased in the nuclear fraction of *VAPA-Rab31*-expressing cells (Figure 3C).

### 2.4. Tumorigenic Effect of VAPA-Rab31 Fusion is Confirmed Using Xenografts

**Figure 4:**
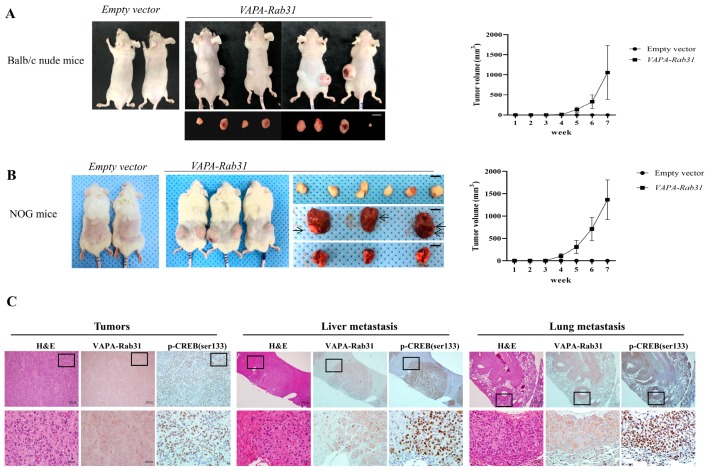
VAPA-Rab31 induces tumorigenesis in the xenograft models. (**A**,**B**) *VAPA-Rab31*-overexpressing cells and control cells were injected subcutaneously to Balb/c athymic nude mice (**A**) and NOG mice (**B**) to compare tumorigenicity. Growth curve of tumors (■) indicated tumor size at the indicated time. Control cells that overexpressed empty vector (●) did not induce tumors in the xenograft mice. Tumors were resected from mice injected with *VAPA-Rab31*-overexpressing cells. In NOG mice, tumors were observed in lungs and liver, as indicated by arrows. (**C**) Tumor tissues were examined by H&E staining. Immunohistochemistry demonstrated overexpression of the HA-tagged VAPA-Rab31 and phosphorylated CREB (p-CREB (ser133)) in tumor cells of subcutaneous tumors, as well as metastatic tumors from liver and lungs.

Furthermore, we examined the effects of *VAPA-Rab31* fusion on tumor growth in vivo using mouse xenograft model in which *VAPA-Rab31*-overexpressing Beas-2B cells were injected subcutaneously. Tumor growth was dramatically increased in mice that were injected with the *VAPA-Rab31*-overexpressing cells (Figure 4A,B). Specifically, control Beas-2B cells did not produce tumors in mice, while *VAPA-Rab31*-overexpressing cells induced subcutaneous tumors in all injected NOG mice as well as the Balb/c nude mice. Furthermore, we found additional tumors in the lungs and liver the *VAPA-Rab31*-xenografted NOG mice. The histologic feature of the tumors was confirmed by hematoxylin and eosin (H&E) staining of formalin-fixed paraffin-embedded (FFPE) tissues that were resected from the mice. Expression of VAPA-Rab31 protein were confirmed by immunohistochemistry using hemagglutinin (HA) tag antibody. Additionally, we observed high expression of phosphorylated CREB by immunohistochemistry in the primary tumors and lung and liver metastases of NOG mice (Figure 4C).

## 3. Discussion

Fusion transcripts of *VAPA-Rab31* have been detected in lung adenocarcinoma and hepatocellular carcinoma (TCGA-LIHC/TCGA-ZP-A9D0). However, the tumorigenicity of the VAPA-Rab31 fusion protein has not yet been investigated. This study demonstrated the oncogenic effect of VAPA-Rab31 fusion in vivo as well as in vitro. Notably, tumor formation in the xenograft mice injected with *VAPA-Rab31*-overexpressing cells was also associated with metastasis formation in lungs and liver.

The somatic mutations that can drive cancer are promising biomarkers and targets for treatment. Gene fusions can also act as oncogenic drivers, and their characterization has been of great importance to identify additional potential candidates [20,21,22]. Gene fusion events can occur by because of an exogenous carcinogenic environment or endogenous factors such as ionization radiation, oxidative stress, and DNA damage. DNA double-strand breaks are mostly repaired by common molecular mechanisms including non-homologous end-joining that can generate gene fusions in the event of erroneous joining [23,24]. We have to identify the specific gene rearrangements that might favor the development of cancer. Cancer specific expression of fusion genes can be a good condition to select oncogenes. In this regard, RNA sequencing in genome level can detect genetic rearrangements in a broad range of tumor types [25,26]. We observed *VAPA-Rab31* overexpression in the tumor tissue from a patient and not in the paired normal tissue. The expression level of *Rab31* mRNA was similar in tumor and normal tissue. However, cells expressing VAPA-Rab31 might be selected and expanded during cancer progression because of a growth and/or survival advantage. Beas-2B cells are immortalized epithelial cells isolated from normal human bronchus and cannot induce tumors in immunosuppressed mice [27]. After ectopic expression of VAPA-Rab31, Beas-2B cells demonstrated enhanced colony-forming activity and tumorigenicity in a xenograft model.

In this study, we found that Rab31-VAPA inhibited apoptosis by increasing the Bcl-2/Bax ratio, consistent with the previously identified function of Rab31 [18]. Overexpression of *VAPA-Rab31* upregulated Bcl-2 both at the mRNA and protein level and made cells resistant to apoptosis. These results show that VAPA-Rab31 contributes to oncogenic condition through inhibition of apoptosis, a known cancer hallmark that plays a role in tumor progression [28].

Wilson et al. have previously reported that the cyclic AMP response element (CRE) site in the *Bcl-2* promoter seems to be a major positive regulatory site for *Bcl-2* expression [29]. CRE-binding protein (CREB), a transcription factor for *Bcl-2* expression, is activated by phosphorylation at serine 133 [30,31]. In this study, we found that tumors and metastases that were induced by VAPA-Rab31 showed strong expression of phosphorylated CREB. Our results therefore suggest that upregulated Bcl-2 and activated CREB could be a potential mechanism underlying the oncogenic effect of VAPA-Rab31. However, further functional analysis is necessary to understand the molecular mechanisms of VAPA-Rab31-directed cancer progression.

In conclusion, we suggest that *VAPA-Rab31* might be a novel oncogenic fusion gene that can be occurred upon genetic rearrangement in lung adenocarcinoma.

## 4. Materials and Methods

### 4.1. Patient’s Specimen and Cell Lines

We investigated the fusion gene *VAPA-Rab31*, discovered from RNA-seq data of a Korean patient with lung adenocarcinoma. RNA-seq data was generated after being approved by the Institutional Review Board (IRB No. NCC-2014-0115) of National Cancer Center Korea (NCCK). Tumor tissue and adjacent normal tissues of the patient were provided by the tumor bank of NCCK.

Human bronchial epithelial cells Beas-2 were purchased from American Type Culture Collection (ATCC) and cultured in airway epithelial cell basal medium using a bronchial epithelial cell growth kit (ATCC, Rockville, MD, USA).

### 4.2. RNA Extraction and RT-PCR

Total RNA was isolated and reverse transcribed into cDNA using the two-step RT-PCR kit (Invitrogen, Carlsbad, CA, USA). To confirm the expression of the *VAPA-Rab31* fusion gene, RT-PCR analysis was performed using primers spanning the breakpoint of the fusion in tumor and adjacent normal tissue of the patient. The fusion junction was amplified using primers spanning the breakpoint (forward primer, 5′-GAACCTAGCAAAGCTGTTCCAC-3′ and reverse primer, 5′-TACATGGGAGCCAATGAATG-3′). The expression of Bcl-2, Bax, and Bak was evaluated by RT-PCR with specific primers. GAPDH was used as quantitative control.

### 4.3. Overexpression of VAPA-Rab31

The full-length ORF of *VAPA-Rab31* was cloned from tumor RNA of the patient, and then inserted into a lentiviral vector with CMV promoter and expressing green fluorescent protein (GFP) as a transduction marker. Lentiviruses of expressing *VAPA-Rab31* were generated from HEK293T cells according to the protocol [32]. Briefly, HEK293T cells were transfected with plasmid mixture including expression vector, lentiviral packaging plasmid, psPAX2, and envelope plasmid, pMD2.G using Lipofectamine^®^ 2000 (Invitrogen). After 48 h, the viral supernatants were harvested and filtered. Virus particles were concentrated for the infection. Beas-2B cells were infected with lentiviruses and stably *VAPA-Rab31*-overexpressing cells were identified because of GFP intensity.

### 4.4. Proliferation and Colony Formation Assay

Beas-2B cells were seeded in 96-well plates (1.0 × 10^4^ cells/well) to assay their proliferation using the IncuCyte™ Zoom system (Essen BioScience, Michigan, MI, USA) according to the manufacturer’s instructions. To test the effect of the VAPA-RAb31 fusion gene on colony formation, cells were seeded in 6-well plates (2.0 × 10^2^ cells/well) and incubated in the appropriate medium, replaced with fresh media every three days. After two weeks, the cells were fixed with 10% methanol and 10% acetic acid and stained with 0.5% crystal violet solution (Sigma-Aldrich, St. Louis, MO, USA). The number of colonies and surface area were measured using the Image J software (National Institutes of Health, Bethesda, MD, USA). Each experiment was performed in triplicates and repeated three times.

### 4.5. Apoptosis Analysis

A proteasome inhibitor, bortezomib (BioVision Inc., Milpitas, CA, USA) was added to the cells for 48 h to induce apoptosis. Harvested cells were fixed in 70% ethanol and stained with propidium iodide (Sigma-Aldrich). Cell cycle was analyzed by flow cytometry (FACScalibur II, BD Biosciences, MD, USA) and the proportion of cells in sub-G1 phase was determined. The cleaved form of Caspase-3 and PARP were examined as apoptotic markers by western blot analysis.

### 4.6. Western Blot Analysis

Proteins were extracted in sample buffer (125 mM Tris-HCl, 20% glycerol, 4% sodium dodecyl sulfate (SDS), and 10% 2-mercaptoethanol), quantified, separated by SDS-polyacrylamide gel electrophoresis, and transferred to membranes. Membranes were incubated with specific antibodies and developed using the electrochemiluminescence method (Amersham Life Science, Buckinghamshire, UK). Antibodies for Bcl-2 and Bax were purchased from BD Science (San Jose, CA, USA) while Caspase-3, CREB, phosphorylated CREB were purchased from Santa Cruz Biotechnology (Dallas, TX, USA). Antibodies against PARP, histone H3, Erk, phosphorylated Erk1/2 (Thr202/Tyr204) and horseradish peroxidase-conjugated secondary antibodies were obtained from Cell Signaling Technology (Danvers, MA, USA). Cytosolic and nuclear fractions were prepared using NE-PER Nuclear and Cytoplasmic Extraction kit (Thermofisher Scientific, GA, USA). Representative data from three independent experiments were demonstrated.

### 4.7. Induction of In Vivo Tumorigenicity

Six-week-old male Balb/c athymic mice and NOG (NOD.Cg-Prkdcscid II2rgtm1Sug/Jic) mice were purchased from Seron Bio (Kyeonggi, Korea). Beas-2B cells (5.0 × 10^6^ cells/0.1mL PBS) expressing *VAPA-Rab31* or the empty vector were injected subcutaneously into the flanks of the mice (*n* = 6 per group). Tumor size and body weight of mice were measured twice a week. Tumor volume was calculated on the indicated days using the following equation: tumor volume = (large diameter × smaller diameter^2^)/2 [33]. Within eight weeks, mice were sacrificed and tumors and organs were harvested for further analysis. Resected tissues were fixed in 4% paraformaldehyde solution and embedded in paraffin. Sections of FFPE tissue were stained with H&E and also analyzed by immunohistochemistry. Briefly, deparaffinized sections were treated with sodium citrate buffer for antigen retrieval, and then incubated with normal serum with 1% bovine serum albumin to block non-specific antibody binding. After incubation with the primary antibodies and biotinylated secondary antibodies, tissues were processed to enzymatic detection with 3,3′-diaminobenzidine substrate. All animal experiments in this study were performed in accordance with the guidelines of the Institutional Animal Care and Use Committee (IACUC) (approval no: KU17167, approved date: 8 November 2017).

### 4.8. Statistical Analysis

Data were compared to test statistical significance by unpaired *t*-Test using GraphPad Prism software (GraphPad Software Inc., San Diego, CA, USA). All reported *p* values are two-sided and significant differences are marked with * (*p* < 0.05), ** (*p* < 0.01), and *** (*p* < 0.005) on figures, respectively.

## Supplementary Materials

Supplementary materials can be found at https://www.mdpi.com/1422-0067/20/9/2309/s1.

## Abbreviations

VAPA	Vesicle-associated membrane protein (VAMP)-associated protein A
Rab31	Ras-related protein 31
CREB	CAMP responsive element binding protein
PARP	Poly (ADP-ribose) polymerase
Bax	Bcl-2 associated X
Bak	Bcl-2-antagonist/Killer
H&E	Hematoxylin and eosin
FFPE	Formalin-fixed paraffin-embedded
