# Supplementary material for: Oncogenic Effect of the Novel Fusion Gene VAPA-Rab31 in Lung Adenocarcinoma
Source: Int J Mol Sci. 2019 May 10;20(9):2309. doi: 10.3390/ijms20092309 (PMC6539523; doi:10.3390/ijms20092309)
Supplement: Supplementary file 1 [file ijms-20-02309-s001.pdf]

Table S1. Fusion variants of Rab31 or VAPA from TCGA database

Cancer (Sample ID)	Fusion Pair	Frame	5' Gene Junction	3' Gene Junction
Breast Invasive carcinoma (TCGA-D8-A147-01A)	DLGAP1_RAB31	In-frame	Chr18:3814059/-1	Chr18:9792151/1
Liver Hepatocellular Carcinoma (TCGA-ZP-A9D0-01A)	VAPA_RAB31	In-frame	Chr18:9937063/1	Chr8:9775275/1
Pancreas Adenocarcinoma (TCGA-FB-AAQ0-01A)	VAPA_METTL4	Out-of-frame	Chr18:9914332/1	Chr8:2547528/-1

Tumor fusion gene data portal from The Cancer Genome Atlas (TCGA) database (www.tumorfusions.org)
